# Critically endangered Rice’s whales (*Balaenoptera ricei*) selectively feed on high-quality prey in the Gulf of Mexico

**DOI:** 10.1038/s41598-023-33905-6

**Published:** 2023-04-25

**Authors:** Jeremy J. Kiszka, Michelle Caputo, Johanna Vollenweider, Michael R. Heithaus, Laura Aichinger Dias, Lance P. Garrison

**Affiliations:** 1 Institute of Environment, Department of Biological Sciences, Florida International University, North Miami, FL USA; 2 Department of Zoology and Entomology, Rhodes University, Grahamstown, South Africa; 3 Auke Bay Laboratories, Alaska Fisheries Science Center, Juneau, AK USA; 4 Marine Mammal and Turtle Division, Southeast Fisheries Science Center, National Marine Fisheries Service, NOAA, Miami, FL USA; 5 Cooperative Institute for Marine and Atmospheric Studies, University of Miami, Miami, FL USA

**Keywords:** Ecology, Behavioural ecology, Stable isotope analysis

## Abstract

Determining the drivers of prey selection in marine predators is critical when investigating ecosystem structure and function. The newly recognized Rice’s whale (*Balaenoptera ricei*) is one of the most critically endangered large whales in the world and endemic to the industrialized Gulf of Mexico. Here, we investigated the drivers of resource selection by Rice’s whales in relation to prey availability and energy density. Bayesian stable isotope (δ^13^C, δ^15^N) mixing models suggest that Rice’s whales feed primarily on a schooling fish, *Ariomma bondi* (66.8% relative contribution). Prey selection using the Chesson’s index revealed that active prey selection was found to be positive for three out of the four potential prey identified in the mixing model. A low degree of overlap between prey availability and diet inferred from the mixing model (Pianka Index: 0.333) suggests that prey abundance is not the primary driver of prey selection. Energy density data suggest that prey selection may be primarily driven by the energy content*.* Results from this study indicate that Rice’s whales are selective predators consuming schooling prey with the highest energy content. Environmental changes in the region have the potential to influence prey species that would make them less available to Rice’s whales.

## Introduction

Understanding predator–prey interactions is critical in ecology, but it remains challenging when investigating highly mobile and elusive species foraging at depth in marine ecosystems^1–4^. This is particularly the case for cetaceans, for which dietary data are often lacking due to the extent of their movements, both horizontally and vertically in the water column, and the lack of stomach content samples or observational evidence of their diets and foraging behavior^5–8^. Obtaining dietary data is, however, crucial to predicting how changes in predator–prey interactions may influence individual and population-level fitness in light of impacts from human activities, including overfishing and climate change^9–12^. With their high metabolic rates, cetaceans rely on predictable prey resources, and changes in prey availability and quality can potentially have population-level consequences, including decreased survival and reproduction rates leading to subsequent population declines^13–15^. Cetaceans exhibit varying levels of foraging specialization, both among and within species and populations, and there is evidence that the cost of living of these marine predators is highly correlated with the energy content of their prey^16^. For example, common dolphins (*Delphinus delphis*) in the northeast Atlantic forage on high energy density prey to meet their energetically costly lifestyle, and disregard prey with lower energy content regardless of their abundance^17^. Predators with high levels of specialization and higher energetic requirements should also be more susceptible to risks associated with the decline of their prey. Information on the feeding ecology and drivers of prey selection are lacking for many cetacean species (including several species on the IUCN Red List of Threatened Species), which is critical information for predicting how populations may be affected by changes in prey availability and quality with increased anthropogenic stressors on marine ecosystems.

Historically, most information on the diets and feeding ecology of baleen whales has been generated from the examination of stomach contents of harvested whales^18–20^. However, this approach is inadequate or inappropriate for a range of reasons, primarily legal and ethical, and stomach content samples from strandings or incidental captures in fishing gears remain rare and sample sizes are often too small. Other approaches, such as the use of multi-sensor (including cameras) suction-cup-attached archival tags have also greatly improved our understanding of the foraging ecology and behavior of large cetaceans^21^. However, deploying these systems on rare and elusive cetacean species can be challenging. Over the past decades, there has been a significant increase in the use of bulk stable isotopes of multiple elements (nitrogen, carbon, and sulfur in particular) from biopsy samples collected on free-ranging whales to assess the trophic interactions and feeding ecology of cetaceans^22–25^. Stable carbon and nitrogen isotope ratios (noted δ^13^C and δ^15^N, respectively) within tissues of a predator reflect those of its prey, providing a useful method for assessing trophic interactions and identifying foraging habitats. Although most studies have investigated patterns of inter-^23,25,26^ and intra-species isotopic niche partitioning^27,28^, there have been increasing efforts to estimate the relative proportions of prey sources in the diets of marine predators using stable isotope mixing models^29–33^. Stable isotope mixing models incorporate uncertainty for each parameter and employ diet-tissue discrimination factors (or trophic enrichment factor, TEF), which account for the changes in isotopes through the food web^29,34,35^. Over the past decade, the use of stable isotope mixing models has greatly increased our knowledge of the trophic interactions and diets of many cetacean species and populations^8,8,34,36^.

Although assessing the diets of cetaceans and other marine predators is crucial in marine ecology, very few studies attempt to understand the drivers of prey choice^17,37^. Why is one or multiple prey items selected more than others? This question is difficult to answer since it requires an understanding of the prey landscape for a given predator, which is often challenging to assess, particularly in open ocean ecosystems. Based on optimal foraging theory, foragers should select prey based on their net energy intake^38,39^. However, in practice, the diets of predators foraging on mobile prey are the result of both prey choice and the effectiveness of anti-predator behavior of prey^38,39^. For large batch-feeding balaenopterid whales, prey profitability should be determined by the energetic costs of lunge feeding, the size and density of prey patches, and the energy content of individual prey species. Actual diets of these whales should therefore be the result of predator habitat selection and resulting prey encounter rates, decisions on pursuing prey, and effectiveness of anti-predator behavior of prey species^39,40^.

The newly recognized Rice’s whale (*Balaenoptera ricei*) is a critically endangered balaenopterid whale (abundance of *ca.* 50 individuals, CV = 0.50; Garrison et al. 2020^41^) endemic to the Gulf of Mexico (GoMex)^42^. Initially identified as Bryde’s whale (*Balaenoptera edeni*), the Rice’s whale seems to have a remarkably narrow core habitat. Systematic surveys carried out by the NOAA Southeast Fisheries Science Center (SEFSC) between 1992 and 2015 suggest that it mostly occurs in a restricted region of the northeastern GoMex in depths ranging from 100 to 400 m^43^. Recent passive acoustic monitoring studies indicate that Rice’s whales also occur persistently in the north-central and northwestern GoMex in similar depth ranges^44^. Although limited, archival tag data suggest that Rice’s whales perform relatively deep dives (150–250 m deep) and forage diurnally, near the benthos on the upper continental slope of their core habitat^43^. There is, however, no direct information on the foraging ecology of Rice’s whales, including how prey choice might be affected by both prey availability and energy density. Here, we investigated the feeding ecology of Rice’s whales in the northeastern GoMex. Specifically, we (1) assessed prey availability using trawl data and (2) investigated prey choice using a combination of stable carbon and nitrogen isotope mixing models and data on energy content of potential prey.

## Materials and methods

### Sampling

**Figure 1:**
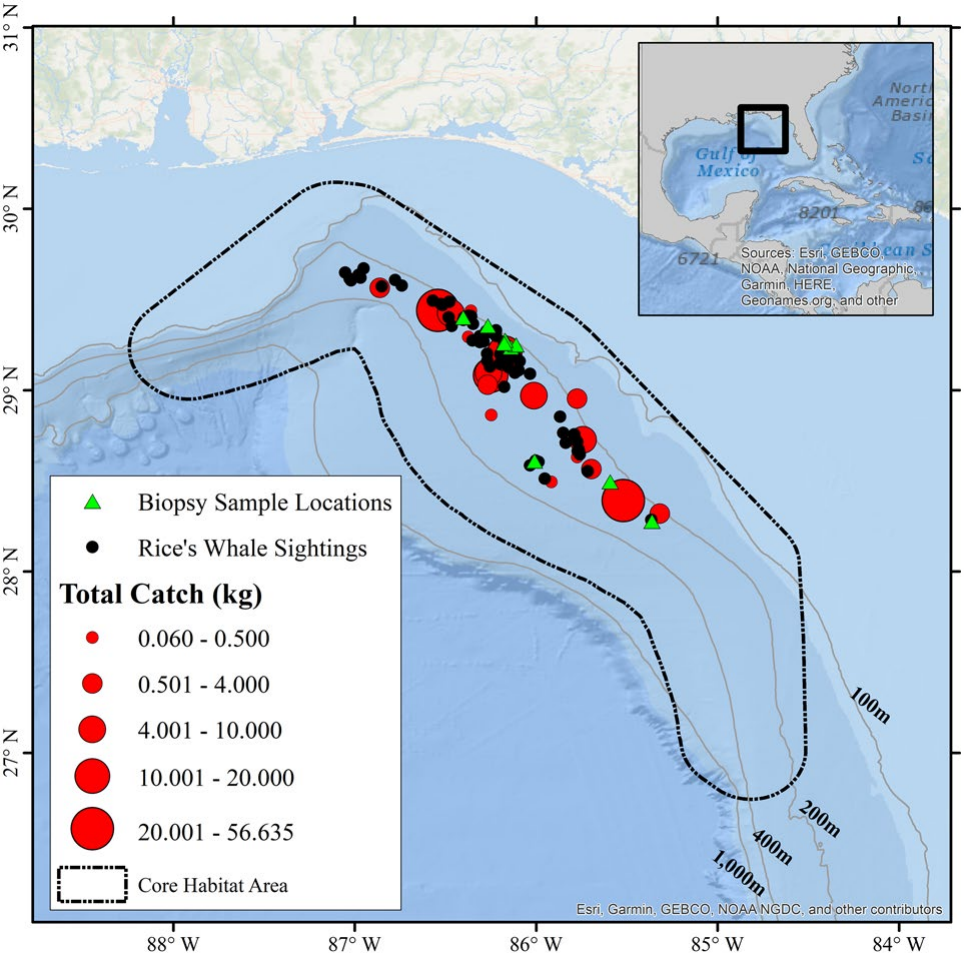
Trawl station locations and total catch (kg) in the Rice’s whale (*Balaenoptera ricei*) habitat in the northeastern Gulf of Mexico collected during July 2019. The locations of Rice’s whale observations during the same survey are indicated along with the core Rice’s whale habitat (Rosel and Garrison^42^). Map created using ArcGIS Desktop version 10.5, ESRI, Inc., http://www.esri.com.

Skin biopsy samples were collected from Rice’s whales during SEFSC research surveys from 2010 to 2019. Biopsy sampling was performed from a 7-m rigid hull inflatable boat with a crossbow fitted with a custom designed sampling dart and head to extract a small core of tissue (7 mm diameter × 40 mm depth). Skin samples were stored frozen at − 80 °C. During the summer of 2019, a mid-water trawl was deployed at selected locations (21 total trawl stations; Fig. 1) during daylight hours to sample potential Rice’s whale prey. Trawl stations were selected by identifying near-bottom aggregations of backscattering organisms based on observations from a Simrad EK80 echosounder (transducer frequencies 18 kHz, 38 kHz and 120 kHz) that was monitored continuously during daylight hours as the vessel surveyed within known Rice’s whale habitat. Once a potential aggregation was identified, the trawl was deployed to target a specific fishing depth, typically 5 m above the bottom at a vessel speed of 3–4 knots (5.5–7.4 km/h). The trawl consisted of a 27 m headrope and *ca.*6 m wing depth with a footrope length of 31.7 m. The liner in the codend consisted of 0.6 cm square heavy delta material. A SimRad ITI gear monitoring system was used to track fishing depth and door spread throughout deployment. The trawl was towed generally near or at the bottom for a maximum of 30 min, not including deployment and retrieval of the net. During all tows, visual observers were stationed on the starboard, port and flying bridge to assist with locating cetaceans that may be in the vicinity to avoid interactions with the trawl. Catches were sorted by species which were then enumerated, measured (total length) and weighed. In each trawl, fish and cephalopods were stored and sampled for stable isotope analysis by collecting a small punch of muscle tissue which were stored frozen at − 20 °C. Whole bodies of selected species were also stored frozen (− 20 °C) for proximate composition analysis. Sampling was conducted by personnel with training and experience collecting biopsy samples from free-ranging cetaceans as authorized by Marine Mammal Protection Act (MMPA) research permits issued by the National Marine Fisheries Service (NMFS) Office of Protected Resources to the SEFSC Marine Mammal Program (MMPA research permit #s 779-1633, 14450 and 21938). Research was also approved by and conducted under the protocols of Florida International University’s Institutional Animal Care and Use Committee (approval IACUC-18-017-CR01). All experiments were performed in accordance to the relevant US guidelines and regulations, and in accordance with ARRIVE guidelines (https://arriveguidelines.org).

### Prey availability

Potential prey species occurrence (%O) was expressed as the number of hauls in which a species was observed, and the relative abundance (%N) was expressed as the number of individuals of a species relative to other species found throughout the series of hauls following methods of Spitz et al.^16^.

$$\% O_{{\text{i}}} = {\text{ n}}_{{\text{i}}} /N \times {1}00,$$

where *n*_i_ is the number of hauls where species *i* was found and *N* is the total number of hauls.

$$\% N_{{\text{i}}} = {\text{ x}}_{{\text{i}}} /X \times {1}00,$$

where *x*_*i*_ is the total number of individuals of species *i* and *X* is the total number of all individuals of all species caught.

Since prey size and weight varied markedly among species, we also expressed the relative abundance of each species in biomass (%B) in hauls:

$$\% B_{{\text{i}}} = {\text{ b}}_{{\text{i}}} /B \times {1}00,$$

where *b*_*i*_ is the total biomass (kg) of individuals of species *i* and *B* is the total biomass of all species caught.

Confidence intervals around prey relative abundances and biomasses were generated using bootstrap simulations, where random samples were drawn with replacement and the procedure was repeated 1000 times.

### Stable isotope analysis and Bayesian mixing model

Stable isotope analysis (SIA) was completed at the Center for Aquatic Chemistry and Environment in the Institute of Environment at Florida International University (North Miami, FL). Rice’s whale skin and prey muscle samples were dried, homogenized into a fine powder, and lipid extracted prior to SIA because lipids are ^13^C depleted^45^. Lipids were extracted by agitating muscle and skin tissues in a 2:1 chloroform:methanol mixture for 1 min with a solvent volume 5-times greater than the sample, after which the samples were left at room temperature for 1 h, centrifuged and the supernatant was removed. After repeating this procedure two more times, each sample was rinsed in deionized water, dried, and 0.4–0.5 mg of sample added to a 4 × 6 mm tin capsule for SIA using a ThermoFinnigan Delta V isotope ratio mass spectrometer (IRMS) coupled with a NA 1500 Ne elemental analyzer. Analytical reproducibility was based on replicates of internal standards including bovine liver (NBS standard reference material) and glycine (Alfa Aesar); variation among standards was 0.07‰ and 0.08‰ for δ^13^C and δ^15^N, respectively. The mean C:N values from analyzed tissues were less than 3, indicating an adequate lipid extraction^46^. Isotopic ratios (R) are reported in the standard delta (δ) notation relative to the international standards of Vienna Pee Dee belemnite (δ^13^C) and atmospheric nitrogen (δ^15^N) using the following equation: δX = (*R*_*sample*_/*R*_*standard*_ − 1) 10^3^, where X is ^13^C or ^15^N and R is the isotope ratio ^13^C/^12^C or ^15^N/^14^N^47^.

Bayesian mass-balance stable isotope mixing models were performed using the ‘MixSIAR’ package in the R statistical programming language^35^ to estimate the relative contribution of potential prey species to the diets of Rice’s whales. Mixing models were run with three Markov chain Monte Carlo chains of 300,000 draws and a burn-in of 200,000 draws. Convergence of the models was checked using Gelmen-Rubin and Geweke diagnostics. Stable isotopes incorporate into tissues based on species and tissue-type specific turnover rates. Since no specific TEF is available for Rice’s whales, we used TEFs from the skin of fin whales (*Balaenoptera physalus*) available in the literature^48^. The TEFs used here were 1.29 ± 0.56‰ for δ^13^C and 2.73 ± 0.58‰ for δ^15^N. The appropriateness of prey groups for Rice’s whale diet and the TEF used here was evaluated by determining the likelihood that prey groups were included in a simulated Rice’s whale mixing polygon^49^. This evaluation method uses a large number of Monte Carlo simulations to create polygons for 1500 iterations of source data (i.e., prey isotope values) corrected using TEFs. It then uses a point-in-polygon algorithm to determine whether a predator’s isotope signature is within or on the edge of the mixing polygon and the proportion of iterations for which consumers fall within the mixing polygon is calculated. It is generally accepted that any consumers falling within the 95% mixing region can reasonably be included in the mixing model, and those outside of this percentile are removed. All statistical analyses were performed in the R statistical programming language v.4.2.1^50^. Mean (± SD) stable carbon and nitrogen isotope data for fish and cephalopod species sampled are provided in Table S1 (Supplementary Material).

### Prey energy density and proximate composition

Fish and squid were thawed and processed in the laboratory at Auke Bay Laboratories (Juneau, AK). Fish length (total length and fork length depending on species) and mantle length of squid was measured to the nearest mm. Whole-body wet mass was measured to the nearest 0.001 g. In preparation for chemical analysis, individual fish were homogenized to a uniform consistency. An aliquot of wet homogenate was dried to a constant mass in a LECO Thermogravimetric Analyzer 701^1^ at 135 °C until a constant mass was achieved. Quality assurance samples dried with each batch of samples consisted of two replicates of Meat1546 homogenate from the National Institute of Standards and Technology, with a maximum standard deviation between the replicates of 0.4 and measured percent moisture within 3% of target values. Dried samples were pulverized to a uniform consistency. Energy density (ED; kJ/g dry mass) of fish was measured using bomb calorimetry. Dried homogenates of 20–60 mg were pressed into pellets and combusted using a Parr 6725 semi-micro bomb calorimeter using standard instrument operating procedures from the instrument manual. Quality assurance samples included with each batch of samples were (1) a benzoic acid standard with each bomb unit within 2% of the target value, (2) a sample replicate with each bomb unit, having a coefficient of variation of less than 1.9 between the replicates, and (3) an internal laboratory standard of homogenized walleye pollock (*Gadus chalcogrammus*) tissue from the Gulf of Alaska within 3% of the target value. Measured energy densities were converted to a wet-mass basis using moisture content.

Lipid content of dried fish and squid was measured using a sulfo-phospho-vanillin (SPV) colorimetric method (modified from Van Handel^51^). Briefly, dry sample homogenates were placed in glass centrifuge tubes with 2 ml of 2:1 (v/v) chloroform/methanol, sonicated for 30 min, diluted to 1:10, and added to a 96-well plate. The plate was heated to 100 °C for 10 min, after which 20 μl of concentrated sulfuric acid was added to each well and heated for 10 more minutes before cooling to room temperature. Subsequently, 280 μl of SPV reagent (1.2 mg/ml vanillin and 80% v/v phosphoric acid) was added to each well and the plate was vortexed at 500 rpm for 30 min. Light absorbance at a wavelength of 490 nm was measured through each sample and total lipid was calculated from a calibration curve. Quality assurance samples included with each batch of samples were (1) two internal laboratory standards of walleye pollock tissue within 8% of the target value, (2) a sample replicate having a coefficient of variation of less than 3 between the replicates, and (3) a blank showing less than 0.00 g lipid. Percent lipid was converted from dry to wet mass basis using moisture content. Protein content was determined from nitrogen measurements. Nitrogen content was measured using an elemental analyzer (FlashSmart elemental analyzer, Thermo Scientific Inc.^1^) following the Dumas method^52,53^. Protein content was estimated using a standard method of multiplying total nitrogen content by a conversion factor of 6.25, the average ratio of nitrogen to protein in animal tissues^54^. Quality assurance samples included with each batch of samples included (1) a blank capsule, (2) an internal laboratory standard of homogenized walleye pollock tissue within 1.5% of the target value, and (3) two sample replicates having a coefficient of variation of less than 0.01 between the replicates. Protein content was converted from dry to wet mass basis using moisture content. Normality was tested using Shapiro-Wilks tests, and %lipid was log transformed to achieve normality, but %protein could not be transformed to achieve normality and therefore a Kruskal–Wallis test was used. ANOVAs were used to test the differences in means between species for energy content (dry and wet) and %moisture. Mean (± SD) proximate composition data for all species sampled is provided in Table S2 (Supplementary Material).

### Prey selection

We used the Pianka index of overlap^55^ to determine whether the composition of Rice’s whales diets, based on isotope mixing models, matched the overall biomass of prey in the core habitat:

$$O = \frac{{\sum {p_{iA} p_{iB} } }}{{\sqrt \Sigma p_{iA}^{2} \Sigma p_{iB}^{2} }},$$

where ${p}_{iA}$ is the percentage by biomass of species *i* in hauls and ${p}_{iB}$ is the percentage by biomass of the species *i* in the diets of Rice’s whales inferred from the stable isotope mixing model. The Pianka index of overlap varies between 0 (no overlap) and 1 (complete overlap).

There is a range of indices to quantify prey selectivity in foragers that have been used for terrestrial and marine organisms, but there is no agreement on which index performs best^56–58^. Here, prey selection was investigated using the Chesson’s index^59^, widely used in a range of foragers to assess prey selectivity^60,61^, including cetaceans^17^:

$$\propto_{i(1 \to m)} = \frac{{r_{i} p_{i} }}{{\Sigma r_{i} p_{i} }},$$

where ${\propto }_{i}$ is the selectivity for prey type *i*, ${r}_{i}$ and ${p}_{i}$ are the proportions of prey *i* in the diet of Rice’s whales inferred from the stable isotope mixing model and from trawl data, respectively, and *m* is the total number of species found in hauls and identified as potential prey in the mixing model. Values of ${\propto }_{i}$ close to 1/*m* correspond to random feeding, whereas values greater than 1/*m* correspond to active selection of prey *i.*

## Results

### Nektonic community composition

**Figure 2:**
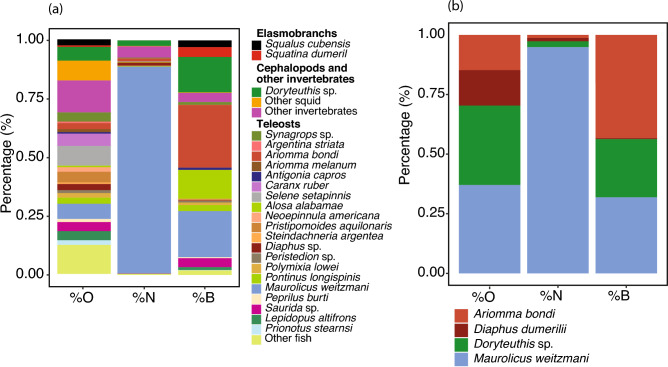
(**a**) Occurrence (%O), relative abundance (%N) and relative biomass (%B) of the nektonic community inferred from trawl surveys conducted in July 2019 and (**b**) of the main potential prey identified for Rice’s whales in the northeastern Gulf of Mexico.

From 21 trawl hauls carried out from 4 to 28 July 2019, a total of 35,598 organisms with an overall biomass of 158.21 kg was collected (Table 1, Fig. 1). Out of 26 total species/species groups sampled, 16 had an occurrence of < 10%. Relative abundance was the highest for *Maurolicus weitzmani* (88.05%, [95% CI 86–90]), but in biomass, two species dominated the nektonic community: *Ariomma bondi* (26.7%, [95% CI 23.9–29.5]) and *M. weitzmani* (19.67%, [95% CI 17.4–22], Table 1, Fig. 2).

**Table 1:** Composition of the nektonic community in the northeastern Gulf of Mexico, where %O is the occurrence; N is the number of fish collected; %N is the relative abundance; 95% CI is the confidence intervals at 95%, %B is the percentage in biomass.

Family	Species	%O	N	%N	95% CI	%B	95% CI
Elasmobranchs							
*Squalidae*	*Squalus cubensis*	19.05	15	0.04	[0–0.2]	2.89	[1.9–4]
*Squatinidae*	*Squatina dumeril*	4.76	1	0.0028	[0–0.1]	4.18	[3–5.6]
Cephalopods							
*Loliginidae*	*Doryteuthis* sp*.*	42.86	819	2.30	[1.4–3.3]	15.1	[12.9–17.4]
Other squids		61.90	61	0.17	[0–0.5]	0.21	[0–0.5]
Invertebrates							
Various		100	1721	4.83	[3.7–6.2]	3.90	[2.7–5.1]
Teleosts							
Acropomatidae	*Synagrops* sp*.*	28.57	122	0.34	[0–0.8]	1.18	[0.6–1.9]
Argentinidae	*Argentina striata*	4.76	5	0.014	[0–0.1]	0.06	[0–0.2]
Ariommatidae	*Ariomma bondi*	19.05	430	1.21	[0.6–1.9]	26.70	[23.9–29.5]
	*A. melanum*	9.52	2	0.0056	[0–0.1]	0.04	[0–0.2]
Caproidae	*Antigonia capros*	4.76	13	0.037	[0–0.2]	0.91	[0.4–1.5]
Carangidae	*Caranx ruber*	38.10	25	0.070	[0–0.3]	0.01	[0–0.1]
	*Selene setapinnis*	61.90	49	0.14	[0–0.4]	0.018	[0–0.1]
Clupeidae	*Alosa alabamae*	4.76	93	0.26	[0–0.6]	12.30	[10.3–14.4]
Gempylidae	*Neoepinnula americana*	14.29	9	0.025	[0–0.2]	0.10	[0–0.3]
Lutjanidae	*Pristipomoides aquilonaris*	33.33	11	0.031	[0–0.2]	0.37	[0.1–0.8]
Merlucciidae	*Steindachneria argentea*	4.76	8	0.022	[0–0.1]	0.17	[0–0.5]
Myctophidae	*Diaphus* sp*.*	19.05	452	1.27	[0.6–2]	0.24	[0–0.6]
Peristediidae	*Peristedion* sp*.*	9.52	10	0.028	[0–0.2]	0.70	[0.2–1.3]
Polymixiidae	*Polymixia lowei*	14.29	73	0.21	[0–0.5]	1.12	[0.6–1.8]
Scorpaenidae	*Pontinus longispinis*	19.05	68	0.19	[0–0.5]	2.53	[1.6–3.6]
Sternoptychidae	*Maurolicus weitzmani*	47.62	31,345	88.05	[86–90]	19.67	[17.4–22]
Stromateidae	*Peprilus burti*	9.52	5	0.014	[0–0.1]	0.40	[0.1–0.8]
Synodontidae	*Saurida* sp.	28.57	55	0.15	[0–0.5]	3.84	[2.7–5.1]
Trichiuridae	*Lepidopus altifrons*	28.57	38	0.11	[0–3]	1.22	[0.6–1.9]
Triglidae	*Prionotus stearnsi*	14.29	13	0.037	[0–0.2]	0.17	[0–0.5]

### Rice’s whale biopsy sampling

A total of 10 skin and blubber biopsy samples were collected on free-ranging Rice’s whales in the northeastern Gulf of Mexico between June 2010 and July 2019. All individuals sampled were physically mature, and in good physical condition (no evidence of emaciation). However, a single sample was collected in 2010, whereas all remaining samples were collected in June and July 2018 and 2019. A multivariate analysis of variance (MANOVA) showed that there was no effect of latitude, longitude, month, or year on stable carbon and nitrogen values (all p > 0.05).

### Trophic interactions and mixing model

**Figure 3:**
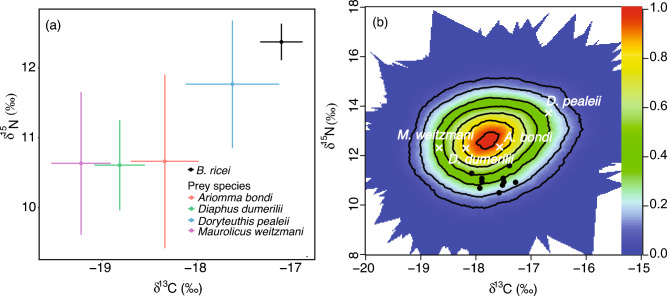
(**a**) Mean stable isotope values for skin samples of Rice’s whale and their potential prey items from the Gulf of Mexico and (**b**) simulated mixing region for the biplot shown in panel (**a**), including the positions of Rice’s whales (black dots) in relation to their prey (white crosses), including the trophic enrichment factor from Borrell et al.^48^ (δC = 1.29 ± 0.56; δN = 2.73 ± 0.58). Contours are used to illustrate probabilities within the mixing region at 5% (the outermost contour) and every 10% interval, illustrated using the color scale and contour lines. The 95% mixing region is the area within the 5% contour.

**Figure 4:**
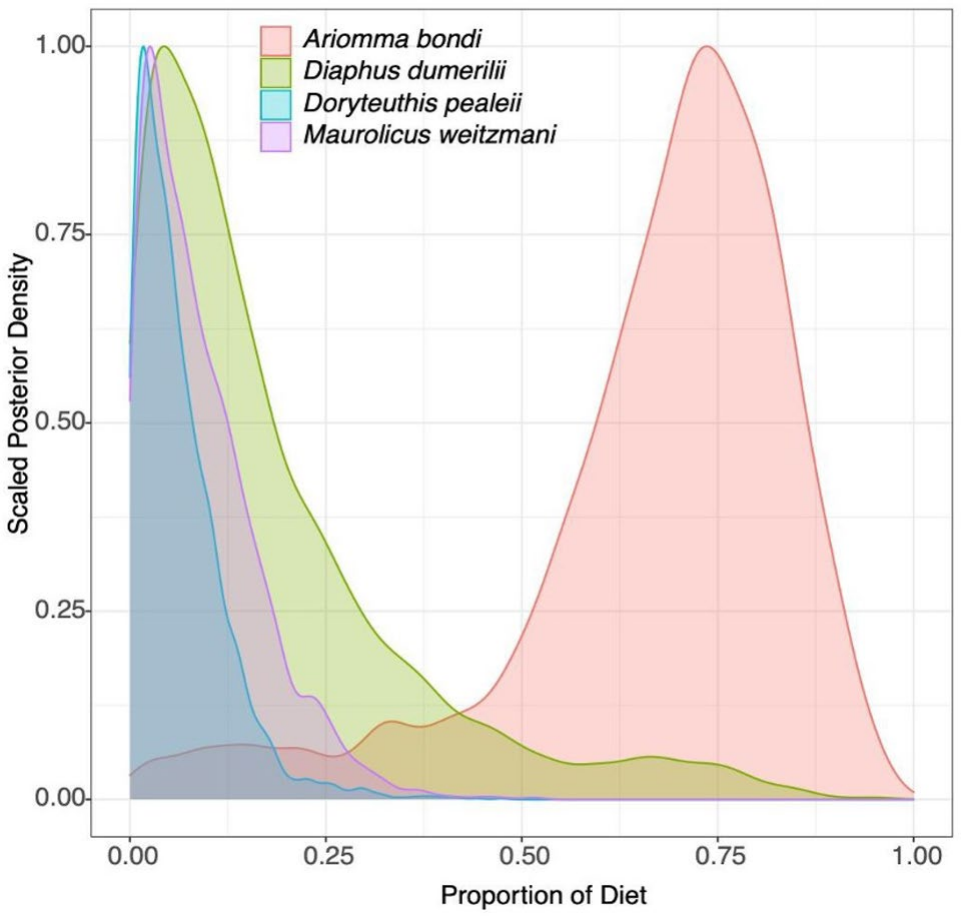
Proportion of dietary contribution of four different potential prey items to Rice’s whale (*Balaenoptera ricei*) diets using Bayesian mixing model analysis with trophic enrichment factors (Borrell et al.^48^) of stable isotope samples taken in the Gulf of Mexico.

Potential prey species (fish and cephalopods) were selected on the basis of their relative abundance in the foraging habitat. Four species largely dominated the nektonic community and were therefore selected for the stable isotope mixing model, particularly *Doryteuthis pealeii*, *A. bondi*, *Diaphus dumerilii* and *M. weitzmani* (Table 1). All Rice’s whale samples (n = 10) fell within the mixing polygon, suggesting that the TEF and prey included in the analysis were appropriate (Fig. 3; Smith et al.^49^). A one-way ANOVA revealed that potential prey included in the analysis had significantly different δ^13^C (F = 20.86, p < 0.0001) and δ^15^N values (F = 12.53, p < 0.01, Table 2). Stable isotope values ranged from 11.98 to 12.88 for δ^15^N (mean = 12.38 ± 0.28) and from  − 17.35 to − 16.82 for δ^13^C (mean =  − 17.09 ± 0.20). Mixing models of dietary contributions identified *A. bondi* as the main prey for Rice’s whales (Fig. 4: 66.8 ± 18.5%), followed by *D. dumerlii* (17.8 ± 17.4%). Other prey had minor relative contributions to the diet of Rice’s whales (*D. pealeii*; 6.4 ± 6.0%; *M. weitzmani*; 9.1 ± 7.5%; Fig. 4).

**Table 2:** Potential prey used in the mixing model to determine prey preferences of Rice’s whale (*Balaenoptera ricei*), including sample size (N), mean (± SD) stable carbon and nitrogen isotope values (i.e., δ^13^C and δ^15^N), trophic level (TL), diet, and habitat.

Species	Common name	N	δ^13^C ± SD	δ^15^N ± SD	TL	Diet	Habitat
*Maurolicus weitzmani*	Atlantic pearlside	18	− 19.22 ± 0.31	10.64 ± 1.02	3.1	Copepods	Pelagic
*Ariomma bondi*	Silver-rag driftfish	7	− 18.34 ± 0.35	10.66 ± 1.24	3.5	Crustaceans	Demersal; 100-200 m
*Diaphus dumerilii*	Dumeril’s lanternfish	8	− 18.83 ± 0.24	10.63 ± 1.27	3.0	Planktonic copepods	Mesopelagic
*Doryteuthis pealeii*	Longfin inshore squid	14	− 17.63 ± 0.49	11.77 ± 0.91	3.4	Small fish, crustaceans, polychaetes, squid	Mesopelagic and benthic

TL, diet, and habitat taken from Fishbase and Sealifebase.

### Proximate composition of prey

**Figure 5:**
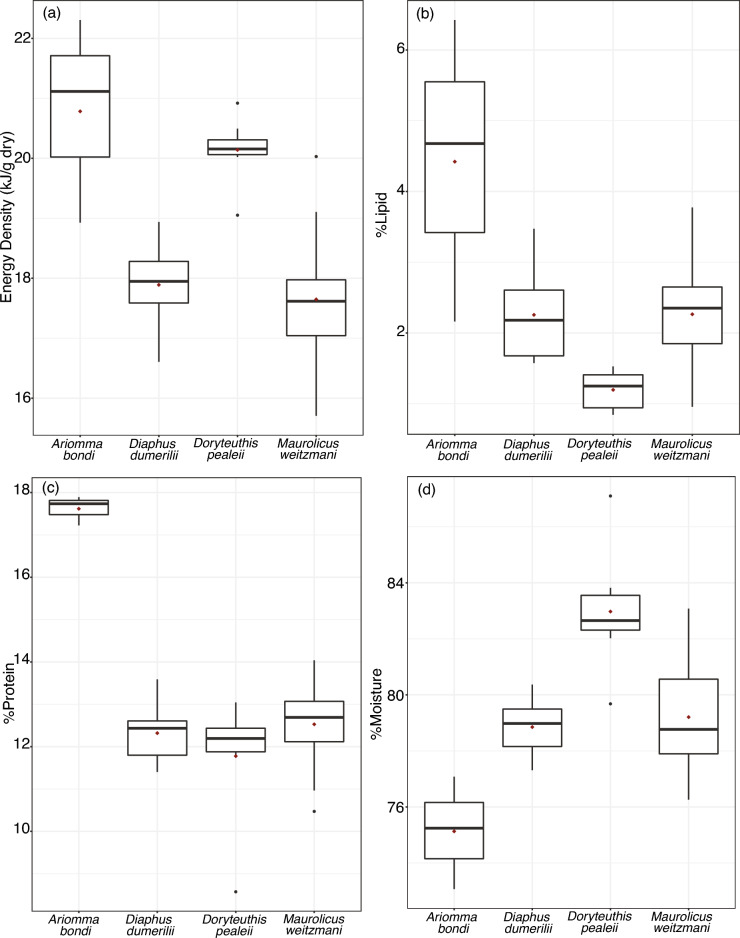
Proximate composition (mean, median, 50, 75 percentiles and outliers) of Rice’s whale (*Balaenoptera ricei*) prey from the Gulf of Mexico, including (**a**) energy density (kJ/g dry), (**b**) %lipid, (**c**) %protein and (**d**) %moisture content.

Energy density (kJ/g wet and dry weights), %lipid, %protein and %moisture was calculated for all Rice’s whale potential prey (Table 3). All species were significantly different (p < 0.0001) for all measurements: energy density (wet: F = 20.82; p < 0.0001 and dry: F = 22.52, p < 0.0001), %lipid (F = 10.40, p < 0.0001), %protein (Kruskal–Wallis: H = 10.11, p = 0.01), and %moisture (F = 17.84, p < 0.0001; Fig. 5). Tukey’s post-hoc testing revealed that *A. bondi* had significantly (p < 0.05) greater energy (wet), lipids, and protein compared to all other species (Table 4). *A. bondi* were also significantly (p < 0.05) enriched in energy (dry) compared to *D. dumerilii* and *M. weitzmani*.

**Table 3:** Energy density and proximate composition of Rice’s whale (*Balaenoptera ricei*) prey from the Gulf of Mexico.

Species	N	Energy density (kJ/g wet)	Energy density (kJ/g dry)	%Lipid	%Protein	%Moisture
*Ariomma bondi*	3	5.15 ± 0.34	20.78 ± 1.71	4.42 ± 2.14	17.627 ± 0.35	75.13 ± 2.01
*Diaphus dumerilii*	7	3.78 ± 0.26	17.89 ± .074	2.26 ± 0.72	12.32 ± 0.73	78.85 ± 1.06
*Doryteuthis pealeii*	8	3.43 ± 0.43	20.14 ± 0.53	1.20 ± 0.27	11.78 ± 1.47	82.97 ± 2.078
*Maurolicus weitzmani*	23	3.67 ± 0.317	17.65 ± 0.94	2.27 ± 0.73	12.52 ± 0.92	79.21 ± 1.71

**Table 4:** Tukey’s pairwise comparisons between proximate composition measures of Rice’s whale (*Balaenoptera ricei*) prey.

	*Ariomma bondi*	*Diaphus dumerilii*	*Doryteuthis pealeii*
Energy density (kJ/g dry)			
*Diaphus dumerilii*	**2.89**		
*Doryteuthis pealeii*	0.64	**2.25**	
*Maurolicus weitzmani*	− **3.14**	− 0.24	**2.49**
Energy density (kJ/g wet)			
*Diaphus dumerilii*	**1.37**		
*Doryteuthis pealeii*	**1.72**	0.36	
*Maurolicus weitzmani*	**1.49**	0.12	0.23
%Moisture			
*Diaphus dumerilii*	**3.72**		
*Doryteuthis pealeii*	**7.85**	**4.12**	
*Maurolicus weitzmani*	**4.08**	0.36	**3.77**
%Lipid			
*Diaphus dumerilii*	− 0.62		
*Doryteuthis pealeii*	− **1.24**	− **0.62**	
*Maurolicus weitzmani*	− **0.63**	− 0.015	**0.60**
%Protein			
*Diaphus dumerilii*	**0.024**		
*Doryteuthis pealeii*	**0.029**	0.0050	
*Maurolicus weitzmani*	**0.023**	− 0.0012	− 0.0061

Bold indicates a significant difference (p < 0.05).

### Prey selection

The Pianka index suggests a relatively low degree of overlap between prey availability (in biomass) and prey consumed (0.333), suggesting that prey abundance is not the primary driver of prey selection. Active prey selection was found to be positive for three out of the four potential prey in the mixing model, particularly *D. pealeii*, *D. dumerilii* and most significantly for *A. bondi* (Table 5). Energy density data also suggest that prey selection may be primarily driven by the energy content of *A. bondi* (Table 4, Fig. 5).

**Table 5:** Values of Chesson’s index (α_*i*_).

Family	Species	α	Selection
Cephalopods			
*Loliginidae*	*Doryteuthis pealeii*	0.039	Positive
Teleosts			
Ariommatidae	*Ariomma bondi*	0.766	Positive
Myctophidae	*Diaphus dumerilii*	0.194	Positive
Sternoptychidae	*Maurolicus weitzmani*	0.001	Negative

## Discussion

Understanding predator–prey dynamics in marine ecosystems and in highly mobile organisms remains a major challenge, particularly in elusive large marine predators such as baleen whales^25,62,63^. This issue is exacerbated when the studied species is rare and critically endangered, and for which minimally invasive approaches should be used to investigate their foraging ecology. This is the first study attempting to describe the feeding ecology of Rice’s whales in their core habitat of the northeastern GoMex, and also the first examination of the potential drivers affecting prey selection in this tropical resident baleen whale species. More specifically, we investigated how prey availability (inferred from trawl surveys) and quality (inferred from proximate composition) would affect prey selection in this predator. Understanding the feeding ecology and prey selection of Rice’s whales is crucial to predict how this species could potentially respond to change in prey availability in the GoMex. Although limited, there is evidence that several species of cetaceans across the globe have experienced population declines due to prey depletion. For example, decreasing abundance of Mediterranean common dolphins has been linked to the decline of small pelagic fish stocks in the Ionian Sea^13,14^. In the coastal waters of the Pacific northwest, southern resident killer whales (*Orcinus orca*) have experienced population declines as a result of Chinook salmon decline (*Oncorhynchus tshawytscha*)^15^. Changes in the spatiotemporal distribution of prey can also have repercussions on life history parameters of cetaceans. As such, reproductive rates of North Atlantic right whales (*Eubalaena glacialis*) can be significantly influenced by prey availability in the Gulf of Maine, and can therefore have major impacts on the recovery of this critically endangered species^64^. The GoMex is exposed to a wide range of anthropogenic impacts that are putting Rice’s whales at acute risk of extinction due to disturbance, collisions with ships, and bycatch^65^. Fisheries and climate change could have severe consequences for Rice’s whale prey and ultimately, the recovery of this species. Therefore, improving our understanding of the foraging ecology of Rice’s whales is important for developing management strategies that will enhance the probability that they are able to persist in this region.

The results of our study might be affected by a number of biases. Stable isotope mixing models are useful to investigate the trophic interactions within food webs, but their precision can be limited, particularly when the predator considered has a broad diet^66^. As such, stomach content analysis remains the most reliable method to comprehensively investigate the diets of cetaceans particularly since it provides the most detailed information on prey composition^17^. Another limitation of our approach to assess the diets of Rice’s whales was the choice of Trophic Enrichment Factor used in our mixing model (TEF for the fin whale was used) because it is the most significant potential source of error in these models^34,66,67^. However, it is generally accepted that TEFs of taxonomically similar species can be appropriate^34,68^. Potential prey selection can also be a significant source of bias. However, we built our mixing model based on the main prey occurring within the Rice’s whale core habitat, which has rarely been achieved in other regions, particularly for other large whales in tropical marine ecosystems. Another limitation of our study is also the absence of a priori knowledge on the feeding ecology of Rice’s whales in the GoMex, or the unavailability of complementary approaches or methods that would have supplemented dietary information from stable isotope mixing models, such as stomach content from stranded individuals, behavioral observations of their feeding behavior, or whale-borne video camera data^31,66^. However, no other sources of information were available, particularly since Rice’s whale strandings are extremely rare, and observations of their foraging behavior are impossible due to the fact that they do not forage at the surface^42^. With regards to prey availability within the Rice’s whale habitat, trawling surveys can be affected by sampling design (spatial and temporal fishing effort, immersion time and depths), and the differential escape capabilities of species sampled^17,69^. Lastly, trawling methods have the tendency to average catch rates over relatively large spatial scales in comparison to the size and distribution of prey patches that Rice’s whales might be targeting. Therefore, it could possibly lead to an underappreciation of the fine-scale mechanisms of distribution and patch densities of Rice’s whale prey, and on how Rice’s whales exploit these prey patches. Despite some limitations, we believe that we used the best methods and practices to reconstruct the diet of Rice’s whales, and to investigate the potential drivers of prey choice.

Our results suggest that Rice’s whales in the northeastern GoMex are selective predators, mostly foraging on high-energy content prey, particularly *A. bondi*. Other prey that are relatively abundant in the Rice’s whale habitat were found to have a lesser contribution. Most significantly, our results suggest that *M. weitzmani*, one of the most abundant species in our samples, was relatively unimportant in the diets of Rice’s whales. *A. bondi* is part of a community of small schooling fish occurring in outer continental shelf waters of the broader GoMex and northwestern Atlantic. It occupies a demersal habitat over muddy bottoms, typically occurs in water depths of 50–500 m, and is described as occurring in large schools^70,71^. Individual *A. bondi* collected during our study ranged in total length from 151 to 200 mm (mean = 168, SD = 9.9, n = 28). Distribution maps from historical catch records indicate that *A. bondi* occur on the outer edge of the continental shelf throughout the northern GoMex^72^. Near-bottom trawl survey data collected over the outer shelf of the northern GoMex between 2003 and 2013 show that this species is common in the GoMex, particularly near the shelf break throughout the north-central and northwestern GoMex (National Marine Fisheries Service, Southeast Fisheries Science Center, unpublished data), where Rice’s whales primarily occur^43^.

Examining prey choices in marine predators such as cetaceans is challenging and difficult to quantitatively assess, particularly in marine environments. However, the results of our study allow us to identify some general patterns in the feeding strategies of Rice’s whales in the GoMex, particularly with respect to the relative importance of prey availability vs. quality. The high importance of *A. bondi*, as suggested by the mixing model, in the diets of Rice’s whales is probably influenced by the relatively high biomass of this prey in the habitat. However, based on proximate composition data and the prey selection analysis, the high energy content of *A. bondi*, relative to other available forage species, likely influences prey selection. *A. bondi* is a schooling species that tends to form dense^70^, and therefore readily detectable, aggregations, which may contribute to their prevalence in the diet of Rice’s whales, as reported in other balaenopterid whales^62,73^. Rice’s whales seem to forage during the day close to the bottom (150-250 m deep), on the upper continental slope of their core habitat^43^, where *A. bondi* seems to also occur predominantly. These deep dives are energetically costly (deep diving, lunge feeding^21^), and whales may need high-quality prey to meet their energetic requirements. Future research should further examine the feeding ecology of Rice’s whales using complementary methods, such as the use of quantitative fatty acid analysis^74^, and if possible, the deployment of whale-borne camera tags that would provide a better understanding of the diet, feeding rates, and foraging behavior of Rice’s whales.

This work also has important management and conservation implications, particularly due to the listing of the Rice’s whale under the U.S. Endangered Species Act where the designation and protection of critical habitat is a requirement. Critical habitat describes the physical and biological features within the area occupied by the endangered species that are essential for their conservation and recovery. The prey resources identified in this study are an especially important component of the Rice’s whale habitat, particularly given their energy intensive foraging behavior and high energetic demands. The apparent reliance on, and selectivity for, a prey species that constitutes the majority of the diet of Rice’s whales makes the protection of *A. bondi* an essential component of protection and conservation strategies for this whale species. This is especially important in light of the high level of industrial activity within the GoMex and the development of new activities such as offshore wind energy and aquaculture. If these new activities have the potential to disrupt the population dynamics or aggregation of Rice’s whale prey, they may indirectly limit the recovery of the species, or even contribute to the extirpation of this species from their habitat. In addition, changes in the oceanography of the region have the potential to influence the population dynamics of prey species or result in shifts in distribution that would make them less available to Rice’s whales. The limited habitat range for both Rice’s whales and their prey make them particularly vulnerable to negative impacts due to climate change. An improved understanding of the physical features that promote both high productivity and aggregations of Rice’s whale prey will help improve predictions of the long-term impacts of climate change on this endangered species.

## Supplementary Information

Supplementary Tables.

## Data Availability

The data for this study is stored at Florida International University and can be made available by the lead author (JJK) upon request.
