# Author Correction: Critically endangered Rice’s whales (*Balaenoptera ricei*) selectively feed on high-quality prey in the Gulf of Mexico

**DOI:** 10.1038/s41598-023-37450-0

**Published:** 2023-06-26

**Authors:** Jeremy J. Kiszka, Michelle Caputo, Johanna Vollenweider, Michael R. Heithaus, Laura Aichinger Dias, Lance P. Garrison

**Affiliations:** 1 Institute of Environment, Department of Biological Sciences, Florida International University, North Miami, FL USA; 2 Department of Zoology and Entomology, Rhodes University, Grahamstown, South Africa; 3 Auke Bay Laboratories, Alaska Fisheries Science Center, Juneau, AK USA; 4 Marine Mammal and Turtle Division, Southeast Fisheries Science Center, National Marine Fisheries Service, NOAA, Miami, FL USA; 5 Cooperative Institute for Marine and Atmospheric Studies, University of Miami, Miami, FL USA

Correction to: *Scientific Reports* https://doi.org/10.1038/s41598-023-33905-6, published online 25 April 2023

The original version of this Article contained an error in the name of author Laura Aichinger Dias, which was incorrectly given as Laura Aichienger Dias.

The original Article has been corrected.

