# Supplementary material for: Critically endangered Rice’s whales (Balaenoptera ricei) selectively feed on high-quality prey in the Gulf of Mexico
Source: Sci Rep. 2023 Apr 25;13:6710. doi: 10.1038/s41598-023-33905-6 (PMC10130045; doi:10.1038/s41598-023-33905-6)
Supplement: Supplementary file 1 — Supplementary Tables. [file 41598_2023_33905_MOESM1_ESM.docx]

**Table S1:** Mean (±SD) stable isotopes values for samples of fish and squid caught during trawling surveys in July 2019 in the northeastern Gulf of Mexico.

| **SPECIES** | **N** | **Length (mm)** | **𝛿^13^C** | **𝛿^15^N** |
| --- | --- | --- | --- | --- |
| *Acanthephyra curtirostris* | 11 | 67.75 | -18.15±0.38 | 8.44±0.78 |
| *Alosa alabamae* | 2 | 265.50 | -20.53±0.3 | 12.25±0.3 |
| *Antigonia capros* | 2 | 142.00 | -18.35±0.1 | 10.99±0.1 |
| *Argentina striata* | 2 | 144.00 | -17.09±0.18 | 14.35±0.18 |
| *Argyropelecus hemigymnus* | 19 | 27.58 | -18.85±0.52 | 8.39±0.44 |
| *Ariomma bondi* | 7 | 168.71 | -18.34±1.24 | 10.66±1.24 |
| *Baldwinella aureorubens* | 2 | 129.00 | -18.4±0 | 12.36±0.11 |
| *Balistes capriscus* | 5 | 12.60 | -19.48±0.35 | 8±1.8 |
| *Bembrops anatirostris* | 3 | 226.00 | -17.82±0.02 | 12.61±0.62 |
| *Benthosema suborbitale* | 17 | 21.71 | -19.47±0.56 | 7.98±0.75 |
| *Bolitaena pygmaea* | 5 | 55.75 | -19.61±0.79 | 7.23±0.87 |
| *Botus* sp. | 1 | 51.00 | -18.69±0 | 7.61±0 |
| *Caranx crysos* | 5 | 17.00 | -19.03±0.27 | 7.93±1.93 |
| *Ceratoscopelus warmingii* | 5 | 48.00 | -19.74±0.6 | 7.8±0.25 |
| *Chauliodus sloani* | 29 | 191.43 | -18.68±0.43 | 9.47±0.36 |
| *Coccorella atlantica* | 19 | 89.53 | -18.5±0.47 | 9.96±0.83 |
| *Coelorinchus caribbaeus* | 1 | 209.00 | -17.44±0 | 14.25±0 |
| *Coelorinchus occa* | 1 | 241.00 | -17.15±0 | 13.97±0 |
| *Diaphus dumerilii* | 8 | 48.88 | -18.83±0.24 | 10.63±1.27 |
| *Dolicholagus longirostis* | 5 | 106.00 | -20.11±0.77 | 7.5±0.71 |
| *Doryteuthis pealeii* | 14 | 105.50 | -17.63±0.04 | 11.77±0.91 |
| *Histrio histrio* | 15 | 23.93 | -17.57±0.72 | 5.43±0.49 |
| *Ilex coindettii* | 2 | 112.50 | -17.94±0.01 | 12.48±0.2 |
| *Latruetes fucorum* | 12 | 12.67 | -16.86±0.58 | 3.97±0.45 |
| *Leander tenuicornis* | 5 |  | -16.88±0.84 | 5.69±0.63 |
| *Lepidophanes guentheri* | 15 |  | -18.89±0.61 | 6.75±1 |
| *Mastigoteuthis agassizii* | 3 | 92.00 | -19.06±0.06 | 12.51±0.69 |
| *Maurolicus weitzmani* | 18 | 42.11 | -19.22±0.09 | 10.64±1.02 |
| *Melamphaes simus* | 15 | 21.93 | -19.54±0.28 | 8.89±0.69 |
| *Myctophidae* spp. | 2 | 43.00 | -19.35±0.08 | 10.35±0.57 |
| *Neoepinnula americana* | 4 | 131.25 | -18.08±0 | 12.75±0.82 |
| *Peprilus burti* | 3 | 169.00 | -17.38±0.1 | 11.63±0.47 |
| *Polymixia lowei* | 4 | 109.75 | -18.19±0.04 | 12.47±0.53 |
| *Pontinus longispinis* | 4 | 178.25 | -17.84±0.03 | 12.78±0.36 |
| *Pristipomoides aquilonaris* | 4 | 194.50 | -17.66±0.05 | 13.1±0.28 |
| *Pterygioteuthis gemmata* | 3 | 10.00 | -20.03±0.31 | 7.79±0.39 |
| *Salpa* sp. | 2 | 124.50 | -20.2±1.38 | 8.09±0.33 |
| *Saurida normani* | 6 | 256.33 | -17.7±0.05 | 13.04±0.69 |
| *Sergia splendens* | 6 | 35.33 | -19.74±0.81 | 6.55±0.5 |
| *Sigmops elongatus* | 15 | 91.20 | -18.89±0.49 | 8.4±1.05 |
| *Steindachneria argentea* | 2 | 230.00 | -17.46±0.01 | 12.75±0.3 |
| *Sternoptyx pseudobscura* | 29 | 25.83 | -19.67±0.29 | 8.92±0.64 |
| *Stigmatoteuthis arcturi* | 4 | 21.25 | -20.5±0.22 | 9.94±1.22 |
| *Stomias affinis* | 26 | 130.20 | -19.38±0.84 | 9.98±0.89 |
| *Synagrops bellus* | 4 | 136.50 | -17.88±0.02 | 11.5±0.24 |
| *Synagrops spinosus* | 4 | 99.25 | -17.67±0.02 | 12.65±0.06 |
| *Synagrops trispinosus* | 11 | 100.91 | -17.84±0.06 | 11.38±1.03 |

**Table S2:** Mean (±SD) proximate composition of fish and squid caught during trawling surveys in July 2019 in the northeastern Gulf of Mexico.

| Species | N | Energy density (kJ/g wet) | Energy density (kJ/g dry) | %Lipid | %Protein | %Moisture |  |  |  |  |
| --- | --- | --- | --- | --- | --- | --- | --- | --- | --- | --- |
| *Alosa alabamae* | 1 | 4.53 | 20.26 | 2.52 | 16.40 | 77.64 |  |  |  |  |
| *Antigonia capros* | 1 | 4.84 | 16.90 | 2.22 | 17.68 | 71.35 |  |  |  |  |
| *Ariomma bondi* | 3 | 5.15±0.34 | 20.79±.71 | 4.42±2.14 | 17.62±0.35 | 75.13±2.01 |  |  |  |  |
| *Baldwinella aureorubens* | 1 | 3.26 | 14.97 | 0.77 | 14.11 | 78.20 |  |  |  |  |
| *Bembrops anatirostris* | 1 | 3.89 | 17.95 | 0.70 | 15.91 | 78.35 |  |  |  |  |
| *Diaphus dumerilii* | 7 | 3.78±0.26 | 17.89±0.74 | 2.26±0.72 | 12.32±0.73 | 78.85±1.06 |  |  |  |  |
| *Diaphus* sp*.* | 5 | 3.08±0.25 | 17.84±1.01 | 2.31±0.33 | 9.43±0.3 | 82.75±0.87 |  |  |  |  |
| *Doryteuthis pealeii* | 8 | 3.43±0.43 | 20.14±0.53 | 1.2±0.27 | 11.78±1.47 | 82.97±2.08 |  |  |  |  |
| *Lepidopus altifrons* | 3 | 3.82±0.36 | 18.93±1.09 | 1.74±0.88 | 14.29±0.26 | 79.82±0.84 |  |  |  |  |
| *Maurolicus weitzmani* | 23 | 3.67±0.31 | 17.65±0.94 | 2.27±0.73 | 12.53±0.92 | 79.21±1.71 |  |  |  |  |
| *Neoepinnula americana* | 2 | 5.42±2.09 | 22.34±3.66 | 9.19±9.19 | 14.02±0.93 | 76.21±5.46 |  |  |  |  |
| *Peprilus burti* | 1 | 5.51 | 22.07 | 4.94 | 15.37 | 75.05 |  |  |  |  |
| *Polymixia lowei* | 1 | 5.57±0.81 | 21.01±2.98 | 3.03±0.05 | 16.68±0.23 | 73.5±0.08 |  |  |  |  |
| *Pontinus longispinis* | 1 | 3.75 | 15.24 | 0.97 | 15.18 | 75.37 |  |  |  |  |
| *Pristipomoides aquilonaris* | 1 | 4.70 | 18.42 | 1.80 | 17.39 | 74.51 |  |  |  |  |
| *Saurida normani* | 2 | 4.72±0.25 | 19.86±0.10 | 1.6±0.89 | 17.82±2.13 | 76.22±1.13 |  |  |  |  |
| *Steindachneria argentea* | 1 | 3.82 | 19.21 | 2.08 | 13.49 | 80.12 |  |  |  |  |
| *Synagrops bellus* | 1 | 3.41 | 16.41 | 0.93 | 12.60 | 79.24 |  |  |  |  |
| *Synagrops spinosus* | 1 | 6.47 | 23.77 | 9.92 | 11.49 | 72.77 |  |  |  |  |
| *Synagrops trispinosus* | 10 | 4.42±0.59 | 19.70±1.27 | 3.98±1.65 | 13.89±0.52 | 77.64±1.64 |  |  |  |  |
